# The Impact of Intimate Partner Violence (IPV) on Parenting by Mothers Within an Ethnically Diverse Population in the Netherlands

**DOI:** 10.1007/s10896-015-9746-2

**Published:** 2015-08-28

**Authors:** Trees Pels, Floor Barbera van Rooij, Marjolijn Distelbrink

**Affiliations:** Free University, Amsterdam, The Netherlands; Verwey Jonker Institute, Utrecht, The Netherlands; University of Amsterdam, PO Box 15780, 1001 NG Amsterdam, The Netherlands

**Keywords:** Family violence, Intimate partner violence, Parenting, Children, Ethnicity, Minorities, Experiences, Care

## Abstract

Intimate partner violence (IPV) profoundly affects multiple life domains for the people involved. We report on the experiences of Dutch mothers of various ethnic backgrounds regarding their parenting during and after IPV, their perceptions of the influence of IPV on their parenting, as well as their need for and experiences with support services. We conducted qualitative interviews with 100 mothers in the Netherlands who had experienced IPV. Most reported negative experiences with parenting (both during and after the IPV), a strong effect of the IPV on their parenting, as well as circumstances that aggravated this effect. The mothers had used multiple sources of formal and informal support. Although most evaluated the support that they had received positively, some also mentioned mixed or negative experiences. Many were still in need of support. Relationships with ethnic background and the severity of IPV are discussed.

Intimate Partner Violence (IPV)—“single or recurrent episodes of any threat or act of mental, physical and sexual types of abuse from a previous or current intimate partner” (Rodriguez et al. 2009, p. 359)—occurs all over the world (Alhabib et al. 2010). Nevertheless, the nature and severity of IPV vary (Graham-Bermann and Edleson 2001), as well as its prevalence (Alhabib et al. 2010). Recent research indicates that lifetime prevalence rates of intimate partner violence (IPV) vary between 11 and 71 % (Alhabib et al. 2010; Archer 2006; Harvey et al. 2007; Garcia-Moreno et al. 2006). Differences in prevalence rates appear to depend both on sociodemographic variables (country of residence, cultural background, socioeconomic position, household composition, and gender), and on methodological issues, including the type of violence studied and research methods (Alhabib et al. 2010; Archer 2006; Harvey et al. 2007; Garcia-Moreno et al. 2006). The experience of IPV has a profound impact on mental and physical health, as well as on quality of life, social, occupational, and parental functioning (Garcia-Moreno et al. 2005; Hungerford et al. 2012; Hedtke et al. 2008; Radford and Hester 2001, 2006; Rodriguez et al. 2009). In this article, we focus on the impact of IPV (of fathers and stepfathers, henceforth called fathers, against mothers) on mothers’ parenting within an ethnically diverse population in the Netherlands, as well as on the mothers’ need for support in parenting or other areas.^1^

In Dutch research, lifetime prevalence rates of IPV vary between 12 % (Wittebrood and Veldheer 2005) and 35 % (Römkens 1992). Wittebrood and Veldheer have analyzed their data for variance according to ethnic background and no differences were found. However, ethnic background might be relevant in understanding differences in IPV’s impact on parenting (Levendosky and Graham-Bermann 2001).

According to the ecological process model of parenting of Belsky (1984), there are three major determinants of parenting: 1) personality and psychological well-being of parents; 2) contextual sources of stress and support, like the marital relationship, social network, and work; and 3) characteristics of the child. The determinants on the different levels influence each other. The probability of effective parenting is highest when all determinants are in a positive mode. As parenting is determined by multiple determinants, a negative outcome in a single determinant may be buffered by the other determinants. The probability of parents functioning competently is lower when more determinants are at risk, especially parents’ psychological wellbeing/ personality.

Previous empirical studies have identified negative relationships between IPV and the psychological wellbeing of mothers (e.g., emotional drain, stress; Hungerford et al. 2012). In addition, IPV can be seen as a contextual source of stress, which might be aggravated by the absence of partner support or even the perpetrators’ undermining of parenting. Empirical studies show that a lack of partner support in IPV situations is a stressor (Letourneau et al. 2011). According to the ‘spillover theory’ of Buehler and Gerard (2002), mothers who are faced with heavy criticism or who are humiliated with regard to their parenting skills are especially vulnerable to lose confidence as parents, and this might negatively influence their parenting (Levendosky et al. 2000; Radford and Hester 2006). The informal network is another source of support that might be at risk due to the IPV. Families in which violence occurs tend to be socially isolated and to harbor a culture of silence (Belsky 1993). The socio-cultural context might also play a role here. For example, several studies have revealed the somewhat paradoxical situation that the emphasis within certain immigrant communities on family collectivism and the mothers’ caregiver role may actually exaggerate the taboo against talking about the violence and seeking support, for instance with leaving a violent husband. This may contribute to the continuation of unfavorable parenting situations (Eldering and Borm 1996a, b; Kasturirangan et al. 2004). With respect to Belsky’s third determinant, child characteristics, there is ample evidence that being raised in the IPV-context might negatively affect children’s physical and mental health, social competencies, and academic performance. Furthermore, IPV might influence how children perceive their parents, e.g., as being less trustworthy and reliable as a role model (Davies and Cummings 1994; Harold and Howarth 2004; Levendosky et al. 2000; Overlien and Hyden 2009; Radford and Hester 2006; Hotun Sahin et al. 2010).

Although child characteristics might be affected by IPV in such a way that they aggravate parenting, several authors suggest that the negative outcomes on psychological wellbeing and stress and support are key factors determining the impact of IPV on mothering (Harold and Howarth 2004; Holden and Ritchie 1991; Hungerford et al. 2012; Letourneau et al. 2011; McCloskey et al. 1995). The lack of support and diminished wellbeing due to IPV can lead to hyper-vigilant, unresponsive, and overly permissive or controlling parenting behavior, as well as to a lack of emotional support for children, insufficient parental protection from witnessing IPV or from becoming a direct victim of the violence. This might lead to less positive parent–child relationships and negative child outcomes (Ballif-Spanvill et al. 2007; Harold and Howarth 2004; Hungerford et al. 2012; Kernic et al. 2003; Letourneau et al. 2011; Radford and Hester 2001, 2006). Research also suggests that the violence can be “transmitted” to children (weak-to-moderate relationship; Stith et al. 2000), although most children do not become either victims or perpetrators when they are older (Dixon et al. 2009). Some studies show that mothers may worry about the long-term transmission of violence to their children, fearing that their daughters will become victims and their sons perpetrators (Autry et al. 2003; Levendosky et al. 2000). However, not all parents appear to be aware of the possible consequences of IPV for their children (Autry et al. 2003).

Contrary to the findings mentioned before, some scholars have reported few differences between the parenting styles of parents who have and have not experienced IPV (Radford and Hester 2001). In some cases, the experience of living through violence might lead mothers to be more empowered or to react with increased care for their children (Levendosky et al. 2000).

Parenting support might help to promote a better relationship between parent and child, as well as to reduce the intergenerational transmission of violence and helping children and their mothers to overcome their suffering (Jouriles et al. 2001; Letourneau et al. 2011). To date, few studies have been conducted on mothers who have experienced IPV with regard to their need for and use of formal and informal types of parenting support. Letourneau and colleagues (2011) describe the needs of mothers with young children from the perception of care providers in Canada. They distinguish needs related to instrumental and informational support (e.g., housing, daycare, and other basic services, as well as information on how to access the care system) from needs related to emotional and affirmative support (e.g., listening, affirmation of the mother’s capacity/skills/confidence for mothering). The service providers identified a lack of intervention programs, training, and other resources to help mothers who have experienced violence to improve their relationships with their young children. Other studies have confirmed the scarcity of evidence-based programs and the lack of knowledge about the effectiveness of programs in this field (Rensen et al. 2008; Sullivan and Alexy 2001).

Even less is known about need for and use of support among mothers of immigrant background. Ethnic minorities may differ from the majority population with regard to the meanings that they attach to violence (Warrier et al. 2002), as well as in their coping strategies. The need for and access to care and parenting support might be affected by culture and processes of acculturation as well (Pels et al. 2006; Rodriguez et al. 2009; Yerden 2008). Parents and young people from non-western backgrounds are known to be underserved (in comparison to Dutch parents) with regard to parental support services, even though they face at least as many problems in childrearing and child development as do parents within the native Dutch population (Pels et al. 2006; Van den Broek et al. 2010).

To help improve support for mothers who have experienced IPV in the care and upbringing of their children, we examine the influence of IPV on the parenting behavior of mothers of diverse ethnic backgrounds in the Netherlands, along with their need for support in parenting and other areas. The study that we describe is based on the following research questions:

1. How do mothers experience their parenting situations during and after IPV, and what influences do they consider IPV to have on their parenting?
2. Which parenting and other support needs do mothers have during and after IPV?
3. How is parenting and the need for support related to ethnic background and the severity of IPV?

## Method

The study described in this article is part of a broader research project in the Netherlands focusing on the parenting experiences of mothers and their children in families facing IPV (Pels et al. 2011; Van Rooij et al. 2015). The data reported in this article are based on extensive qualitative interviews with 100 mothers on their parenting experiences and their need for support in parenting and other areas.

### Ethics

We obtained approval from several municipal and national funding organizations for the study. An expert group (including clinicians, policy makers and researchers) was installed to reflect on important decisions regarding the research, findings, and implications for policy and practice. All participants received verbal and written information about the study and consented to participate in the study. They were assured confidentiality and anonymity, and they were informed about the possibility to withdraw during the interview.

We took several measures to ensure the safety of both participants and interviewers (e.g., discussing with mothers possible risks before joining the study; discussing a safe location for the interview). The mothers were also offered the opportunity to contact the interviewer after the interview, if needed, or were referred to the local Domestic Violence Support Points for help.

### Recruitment

We used several methods to recruit the mothers. Our recruitment took place in the four largest cities in the Netherlands (and their outskirts). In all, 178 key individuals (e.g., professionals working in the field of IPV, parenting, youth or community services, and professionals or laypeople working with or having a wide network within the targeted communities) helped us to find potential participants. In addition to these key individuals, we used leaflets, a website about the study, radio advertisements, ads on relevant websites, and a social media site about youth and IPV to help with the recruitment. Furthermore, we asked participants whether they knew of others who might be willing to participate.

Inclusion criteria for mothers in the broader study were: 1) mothers who had experienced IPV (for the last time between 3 months and 6 years before the time of the interview), 2) having at least one child (in the age range preadolescent to young adults: 9–25 years) who was also willing to be interviewed for the broader study. As finding mother-child couples was rather difficult, we also allowed mothers to enter the study without their children. We explicitly aimed at recruiting mothers of various ethnic background (i.e., native Dutch, as well as Turkish, Moroccan, Cape Verdian, Antillean, Afro-Surinamese, Hindustani-Surinamese, Ghanaian, and other ethnic backgrounds). The specified non-native groups account for the largest non-western ethnic groups in the Netherlands. During the process of recruitment and data collection, attention was paid to sufficient inclusion of often less researched categories such as lower educated mothers and non-western mothers. To reach this aim, recruitment took place through many channels, of which key figures from informal organizations for ethnic minorities or women, or from formal support organizations were most fruitful. No incentives were provided. The final sample for this study included 100 mothers.

### Instruments and Procedure

The first version of the interview guide was based on three sources. The first source were reviews of Lünnemann et al. (2011) and Lünnemann and Pels (2013) on the existing literature on IPV, parenting, and the inter-generational transmission of IPV. The second source of information consisted of interviews with caregivers and other key individuals within the various ethnic communities. The final source consisted of previously constructed questionnaires regarding parenting in immigrant families (Pels 2000), youth and IPV (Lamers-Winkelman et al. 2007; Skinner et al. 2005), and the intergenerational transmission of violence (Dijkstra 2000). We also consulted several experts and interviewers of various ethnic backgrounds with regard to the completeness, relevance, and comprehensibility of the topics. We tested a preliminary version of the interview guide in a pilot study, after which we made minor changes (largely concerning the sequence of questions).

The interview guide consisted of an introduction and a semi-structured questionnaire regarding socio-demographic background variables, followed by open-ended questions about the history of IPV and the ways in which the children had experienced the violence; parenting in general, as well as during/after the period of violence; other problems affecting parenting; the influence of violence (and parenting within this context) on children; the intergenerational transmission of violence, and the mothers’ need for and experiences with support in parenting and other areas. A final section consisted of a debriefing, including an (open) evaluation of the interview and a discussion of the possibility of follow-up care (if desired by the mother). Throughout the interview guide, examples were given of how to elicit detail in cases where answers were brief or needed clarification.^2^

Based on the interview, the severity of IPV was classified (by the interviewers) as light (scarce violence, with minor or no injuries and moderate violent behavior or psychological violence), moderate (repeated violence over the year, with occasional minor to moderate injuries, psychological violence), serious (repeated monthly violence often resulting in serious injuries, psychological terror), or very serious (weekly to daily violence, sexual violence, uncontrolled violence combined with verbal humiliation, within a context of threat, control, and fear, psychological terror). The classification was developed and operationalized for the purpose of the current study^3^ in interaction with Dutch experts on IPV. In a minority of cases, interviewers were unsure about the degree of severity. Therefore in the analyses, IPV was recoded into two categories: light/ moderate versus severe/very severe.

Interviewers also completed a form about the interview conditions (duration, location, language use, circumstances, presence of other individuals and their influence, course of the interview, and attitude of the respondent), in order to assess their possible impact on the quality of the interviews. Conditions were not always optimal, the main problem being that, once talking about the violence, it sometimes was not easy to direct the interview to the topic of parenting. However, all interviews provided important information, although the amount of information sometimes differed by topic.

We collected the data through face-to-face interviews. The mean length of the interviews was 1.75 h, and audio recordings were made for 93 % of these interviews. The interviews were conducted by a pool of 20 specially trained interviewers, aged 25–45. With one exception, all of the interviewers were women. The team was mixed according to ethnic background. Ethnic matching was practiced based on the preferences of the interviewees. The interview locations were also selected according to the wishes of the interviewees (e.g., in the home, at the workplace, or in a school or neighborhood center). The interviewers received extensive training focusing on interview techniques, safety issues, cultural and inter-personal sensitivity, and knowledge concerning IPV. During the course of the research, feedback on the interviews was provided by the researchers individually and in group sessions.

### Analyses

All of the interviews were transcribed verbatim (in some cases, they were translated by the interviewers). The quantitative data regarding socio-demographic background and history of IPV were entered in SPSS and analyzed using descriptive statistics. The process for analyzing the open-ended questions was based on the Constant Comparative Theory (see Boeije 2002) and comprised multiple stages. It should be noted however that we only started in-depth coding and analyzing after the data was collected. The first stage consisted of the thorough reading of a selection of the transcripts by four researchers. A coding system was developed based on the interview guide and refined according to the findings from the reading sessions. This coding system was refined through several rounds of feedback in a next stage. In this process, some of the transcripts were coded manually by seven researchers using the developed coding system. These researchers then discussed differences in the attribution of codes, and refined the codes and the coding process together based on consensus. All of the transcripts were subsequently entered and coded in Atlas Ti, a computer program designed to help structure the process of analyzing qualitative data. In this program, several codes can be attached to any given citation (or part thereof). Five researchers were involved in the coding process, each coding a substantial portion of the transcripts.

Coded segments of the interviews were printed and distributed thematically over four researchers. Each selection was sorted by ethnicity and other relevant background variables (e.g., severity of IPV). Each printed coded segment contained coded information on the background variables: age, ethnic background, educational level, family composition, the severity of IPV and a short memo summarizing the entire interview. Researchers analyzed and categorized the evidence for each code by reading and rereading the printed output. During this process, specific attention was paid to differences based on ethnicity and other background variables. The researchers regularly met to talk through and compare their analyses on individual cases as well as on an aggregated level.

## Results

The findings presented here are organized in three main sections: 1) characteristics of the sample and mothers’ evaluation of the interview; 2) parenting (subsections: parenting during and after IPV and the perceived relationship with IPV, parenting practices related to coping with consequences of IPV, differences in parenting related to ethnic background and severity of the violence) and 3) support (subsections: seeking support, informal and formal support experiences, current support needs, barriers and motivators to help-seeking; where relevant, we mention the ways in which ethnic background and severity of IPV relate to support).

### Sample Characteristics and Mothers’ Evaluation of the Interview

Table 1 contains a description of the socio-demographic background of the 100 mothers participating in this study. The mothers varied considerably with regard to their experiences with IPV; we classified 48 % of the cases as light/moderate and 52 % as serious/very serious. Antillean mothers appeared to be under-represented among the groups having experienced serious or very serious violence.

**Table 1:** Socio-demographic background of mothers (*n* = 100)

Variables	Mothers
Age in years - *M* (*SD*)	40.36 (8.29)
Ethnic background – n (%)	
Dutch	14 (14 %)
Antillean/ Aruban	19 (19 %)
Afro-Surinamese	7 (7 %)
Turkish	15 (15 %)
Moroccan	18 (18 %)
Hindostani-Surinamese	15 (15 %)
Capeverdian	5 (5 %)
Other	7 (7 %)
Educational level^ - n (%)	
Low	47 (47 %)
Intermediate	37 (37 %)
High	14 (14 %)
Other	2 (2 %)
Family composition	
Number of children - *M* (*SD*)	1.98 (1.04)
Single - n (%)	79 (79 %)
Dutch language skills (*N* = 86)-N (%)	
Never problems	57 (66 %)
Difficulties with getting by financially	41 (41 %)

Most mothers evaluated the interview positively, mainly because they were given the chance to tell about their experiences and emotions. One fifth had mixed feelings, because the interview had stirred up the painful events they would prefer to forget. None of the mothers discontinued the interview.

### Parenting

#### Parenting During IPV and the Perceived Relationship with IPV

Ninety-three mothers discussed their own parenting practices during IPV. One in five of these mothers reported that they had not always succeeded in protecting their children from the violence (or from witnessing it) during the period of IPV. Two typical answers were, “It is impossible to protect them from harm,” and “We gave them an anxious and insecure situation.” Most of the mothers did mention strategies that they had adopted as a way of shielding their children. In order of frequency, from high to low, they mentioned: avoiding quarrels in general or in front of the children, or sending the children away when quarrels began; guarding children against violence; hiding the violence (or its emotional and physical impact); fleeing or leaving the partner; paying attention to children; and calling on external help (ranging from the police or social services to spiritual help or help from God).

About one in ten of the mothers mentioned that, despite the circumstances, they had succeeded in being “good” (or good enough) mothers during the IPV period. Nevertheless, they also mentioned that it had required considerable energy, and that they had not always been able to hide their emotions (e.g., sadness, depression) from their children. The majority (three out of five), however, mentioned that IPV had exerted a negative influence on their parenting. Due to their own (mental) health problems as a consequence of the IPV, one out of six mothers mentioned *a lack of attention* to their children. They reported that they had lacked the energy necessary to spend quality time with their children, or even to be nice to them: “I only gave her food and clean clothes . . . the remaining time I only cried”; “I did not have the strength to be nice to my children”; “I was functioning on autopilot.” Moreover, one out of seven mothers reported considerable *negligence*, bordering on outright neglect. These mothers mentioned that they had sometimes been unable to find the strength to look after their children, to clothe and feed them, or to take them to school. About one fifth of the mothers mentioned that they had sometimes *taken their own frustrations and peevishness out on their children* by nagging and scolding (“The moments I couldn’t shout at him [father], at such moments I could shout at my children”), and by beating (“When I was hit, then, eh, I also hit my daughter. I passed it on to my daughter”). Several mothers reported that they had later regretted having lost control. As stated by one mother, “Hey, what am I doing now, if I do the same as he [father] does, I act like him.” A few mothers reported that they had *been curt with their* children in an attempt to prevent their behavior (e.g., being loud or disobedient) from igniting irritation and new violence by the fathers. In contrast, one in seven mothers reported that they had *spoiled* their children or that they had been overly indulgent in an attempt to compensate for all of the trouble that the children were experiencing, or because of feelings of guilt: “Actually, he had to be corrected, but I didn’t do that at all. I tried more to handle Dad’s aggression, and to compensate for that.” One in ten mothers also mentioned that *their children had taken over adult or parental responsibilities* or tasks (parentification). Examples included children who had called the police, protected their mothers or functioned as confidants, or taken care of younger children or the housekeeping. Some mothers mentioned difficulties associated with teaching their children one thing while acting differently due to the IPV:

I taught my children to lie. I realized that I really gave a bad example for a long period, but I did not have a choice. If I wanted to continue seeing my family, I had to lie. And they had to as well. They also wanted to keep seeing their grandfather, grandmother, or uncle. I did tell them that lying was very bad. But that we had to do this, because Dad would make a fight. (Turkish, 41, lower education)

Two thirds of the mothers perceived that their partners had not positively contributed at all to parenting. A third of the mothers mentioned aggression, humiliation, and power displays of the fathers toward their children.

#### Parenting after IPV

Eighty-five mothers discussed their parenting practices after IPV had come to a halt. Mothers were more positive about their parenting after the IPV period than during the IPV period. One third evaluated their current parenting situation positively (vs one tenth during the IPV period). They mentioned several improvements, including that the tension and aggression in the family had subsided; that peace and quiet had returned; that they had more time and attention for their children; and that they had more room for doing pleasant things together. Some of the mothers also mentioned the freedom that they now had to raise their children in the way they wanted or to go wherever they wanted with them. They also mentioned their increased confidence, the improved relationships that they had with their children, and the improved structure in their parenting. Nevertheless, about two third of the mothers evaluated their current parenting situation as a mix of both negative and positive aspects or only as negative. Part of these mothers reported to be currently unable to cope with raising their children, either sometimes (one fifth) or continually (about one tenth).

Among the group that reported mixed experiences, the positive experiences that were mentioned resembled those reported by the “positive only” group. The negative experiences with parenting that mothers in the “mixed group” and the “negative only” group reported related to three factors, which we describe successively.

- Problem behavior of their children. About a third of the mothers admitted to having had problems dealing with problem behavior of their children. Twenty-five mothers reported that their children were having internalized problems, sometimes in combination with externalizing problems. These mothers were concerned about their children and/or did not know how to handle them. Eighteen mothers reported having problems related to the externalizing behavior of their children and/or having control over their children (e.g., children not listening to their mothers, not following rules, being rebellious or aggressive). Some of the mothers related their children’s problems to their experiences with IPV in the past, to a lack of structure, or to indulgence and overcompensation during the period of IPV. Others related such problems to the children’s current lack of the strong hand of their fathers.
- Personal circumstances resulting in difficulty coping as a parent. Additional circumstances that were reported as having a negative influence on their parenting included that mothers had felt their mental health to be faltering. About two thirds of the mothers mentioned that they had experienced internalizing problems like sadness or depression in response to the IPV. Some also reported that they had thought about or attempted suicide. Other feelings that were mentioned included anxiety, helplessness, negative self-image, and low self-confidence. Two fifths of the mothers reported health problems, often related to their experiences with IPV. About the same number had experienced financial problems.
- Problems related to the children’s father. A third negative influence on parenting, as reported by the mothers, involved their continued contact with the father of their children. About half of the mothers, including those who were still living with the fathers, were critical or predominantly negative about the behavior of their children’s fathers. In most cases, they complained about the *quality of parenting* by the fathers (in order of frequency): lack of parenting skills and responsibility; aggression or humiliating behavior; carelessness; lack of involvement; instilling a negative image of the mother; poor contact with the children; stimulating machismo in sons, and restricting the freedom of daughters. In addition, mothers complained about *the fathers’ access to the children*. They reported that they had encountered legal problems concerning the arrangements for contacts between father and child, that fathers had not kept their appointments with the children, that their children had been unwilling to see their fathers, continued to have feelings of danger/insecurity, and exhibited difficult behavior after seeing their fathers. About a fifth of the mothers reported that there was no longer any contact with the fathers due to these types of problems.

However, a substantial portion of the mothers also reported positive evaluations of their current contact with the father. The children of these mothers had frequent contact with their fathers and were always able to call them, and the fathers helped when asked, accompanied the mothers to important meetings, or helped in disciplining the children.

#### Parenting Practices Related to Coping with Consequences of IPV^4^

In discussing their parenting after IPV, some of the mothers (*n* = 85) referred to their strategies to help their children cope with the consequences of the violence, including talking with them about how to avoid becoming victims or perpetrators themselves, comforting, paying attention, and providing peace and structure. Two out of five mothers explicitly reported that they talked with their children, with the hope of preventing them from following the example of their parents (i.e., to prevent violence from being repeated in the next generation). To this end, their communications with their daughters tended to emphasize the importance of equality for women, assertiveness, financial and other forms of independence, as well as the necessity of being selective in choosing a partner. To their sons, they stressed the importance of respect, empathy, and caring in their relationships with women and children. In some cases, however, this mission was sometimes frustrated by the fathers, as illustrated by the following quote:

But at my ex [his father’s place] it is again like “a man is everything, and a girl is just nothing.” A girl has no opinion and has nothing to say; she only has to accept and carry on, and humbly do everything and shut her mouth. (Moroccan, 47, lower education)

Mothers also talked about the IPV to inform their children about the context of the IPV, to emphasize that the violence had not been the children’s fault, and to teach them that IPV is not normal. In general, the mothers had noticed that children were not eager to talk about what had happened or about their feelings in relation to the situation, although the mothers did try. When their attempts were unsuccessful, they tried to persuade their children to talk with others (e.g., family, professionals, or peers in similar situations). Some mothers reported that they did not talk with their children about the IPV because they were afraid that doing so would revive or aggravate their children’s emotions.

#### Relationship with Ethnic Background and Severity of the Violence

Regarding the IPV period, we did not find clear differences between ethnic groups with regard to the mothers’ reports concerning the negative influence of IPV on parenting and the protection strategies they employed. We did, however, observe differences regarding the period afterwards: The Hindustani mothers were the most positive, whereas the Turkish mothers reported the most negative experiences with parenting. Mothers who had faced severe violence reported more negative parenting experiences both during and after the IPV, compared to the other mothers. Mothers who had experienced violence of greater severity were more likely to report that they had been unable to shield their children. These mothers reported having adopted such strategies as avoidance, hiding the violence, and fleeing or leaving their partners. With respect to the support by fathers, Turkish, Hindustani-Surinamese and Moroccan mothers expressed the most negative views. Dutch mothers were most likely to report that fathers had exhibited serious violence towards the children (three out of five), followed by Turkish mothers (about half). Antillean mothers were the least likely to report serious violence towards the children. Mothers who had experienced violence of greater severity were more negative about the parenting practices of fathers.

### Support from the Informal and Formal Network

#### Seeking Support

In all the interviews, support seeking and finding was discussed. Isolation (due to e.g., recent migration, active shielding by partner or in-laws), shame, the desire to avoid hurting the family’s honor, a negative attitude towards divorce, and threats by partners appeared to have kept a substantial proportion of the mothers from talking about the violence and seeking help (either during a certain period or throughout the entire IPV period). For the women who had migrated relatively recently, issues related to immigration (e.g., a small family network in the Netherlands, problems with finding their way) also played a role:

Back then I didn’t know anyone. That was what I missed most. I didn’t have anyone here. I had to ask my mother-in-law everything. And she was very precise. I had to clean and cook the whole day. I wasn’t any good for anything else. They brought me here as their servant. You know, I had to take care of their grandmother for 7 years. (…) If I would have been in Turkey, they would never had been able to do all this to me. I would have had the support of my parents and brothers. Here they humiliated me.. Here I suffered. I did not have anyone… in my time, you did not leave your husband. You had to endure what was being predestined for you. That is your faith. (Turkish mother, 46, lower education)

Often inspired by a negative attitude toward divorce within certain ethnic communities, the high pressure that some relatives exerted on women to remain married was also frequently reported as a reason why some mothers had not sought support. This was particularly common among Turkish, Moroccan, and Hindustani women. Mothers of different ethnic backgrounds also referred to the taboo on IPV and on talking about private issues (e.g., “Don’t air your dirty laundry in public”). Despite the high pressure to stay married or to hide problems, some mothers had simply done what they needed to do in order to end their hazardous situations:

No, you had to endure/keep up for the people. And then I said, “Stop,” because I had had it. For the people? As long as the people don’t pay my rent, give me food, as long as the people didn’t see my life, what it looks like; I don’t stay with him for the people. I have to think about me and the children; they need peace. They don’t need their mother having a black eye or a blue neck or a knife at her throat. (Moroccan mother, 35, intermediate education)

#### Informal and Formal Support Experiences

Mothers had been in contact with several sources of support, both informal and formal. Eighty-five mothers discussed their experiences with informal support during the IPV period. Mothers often first sought informal support. One out of five was positive, one tenth had negative and two third had mixed experiences with family members, related to the taboo on family violence and on divorce. In many cases, however, these relatives had changed their minds, usually after the violence had ended, and they started supporting the mothers more, apologizing for not having been a mainstay sooner. Relatives were generally regarded as a positive source of practical support (e.g., helping to find a house, taking care of the children), and emotional support. Experiences with in-laws were diverse, ranging from highly supportive to still conspiring with their husbands (or former husbands). About half of the mothers reported receiving support from their in-laws, whereas the other half had negative experiences. Mothers-in-law were especially influential among Turkish, Moroccan, and Hindustani mothers, particularly for those who had recently immigrated. Few Antillean and Afro-Surinamese mothers mentioned their in-laws (either positively or negatively) with regard to support. About three fourths of the mothers mentioned other informal sources of support, including neighbors, friends, acquaintances, the ethnic community, churches or mosques, fellow sufferers, and new partners. In addition to positive support, some mothers referred to a lack of understanding on the part of representatives from these categories.

The stories about formal support varied widely, with respect to the organizations with which the mothers had been in contact, as well as in terms of their experiences. More than half of all mothers interviewed had been in contact with youth-service organizations, and about the same number had been in contact with psychologists, psychiatrists, and personal coaches. Almost half of the mothers had been in contact with the police and two fifth with professionals at schools or daycare centers. One third of the mothers had been in contact with social workers, one third with general practitioners and/or hospitals, and one fifth had stayed in women’s shelters. In general, the mothers appeared satisfied with the help they had received from formal care institutions. Positive experiences were often related to easily accessible forms of parenting support and the supportive role of schools for their children, as well as for themselves.

Ehh, if you say organization, school I trust, but outside school—a parenting specialist—I have little confidence. Why? With them, I have to make an appointment, but at school, I see them every day. I do trust school more. (Turkish, 40, lower education).

Negative experiences were reported as well. For example, mothers referred to negative experiences with support that did not correspond to their specific needs, that was not appropriate to their situations, or that was unsatisfactory in terms of effectiveness. Explicit negative experiences related to support within the context of enforcement (e.g., guardianship, children and family court advisory and support service, and the Child Maltreatment Advisory and Contact Point). In some cases, mothers felt trapped between the demands of the various institutions that they were obliged to obey. For example, one institution demanded that mothers ensure that their children are safe at home (under the threat of having their children removed from the home), even though Dutch law requires that mothers allow their children to have contact with their (aggressive) fathers. In another example, professionals insisted that mothers separate from their husbands, even if this is not an option because of the taboo on divorce within the mothers’ communities.

#### Current Support Needs

At the time of the interview, more than half of the 58 mothers who discussed their current need for support had no desire for support. Among them were also mothers who reported to be in need of support but expressed no desire to actually seek help. They did not wish to share their problems with outsiders or were afraid to do so because of negative experiences with formal interventions or institutions. Examples of these negative experiences were: no adequate protection by the police when they had finally decided to report their partners’ behavior or fled their homes with their children, or a fear of losing their children if they were to seek help as a parent.

The mothers who did wish to receive formal help primarily sought practical help with rebuilding their lives (e.g., finding a house, finding daycare for their children so they could work or study, securing financial support, or finding work or an educational program). They also wished to have support with raising their children. In part, the support that they desired had to do with issues related to IPV (e.g., help with talking with children about the consequences of the violence), although it was also partly related to other issues as well (e.g., single parenthood or helping children to cope with a handicapped sibling). Some mothers expressed a desire for couples therapy. Moroccan mothers were particularly likely to express a desire for formal help.

#### Barriers and Motivators to Help-Seeking

Reflecting on their experiences, the mothers discerned several barriers that had kept them from seeking support. These barriers included psychological problems (which made it more difficult for them to seek help), financial barriers (because of their dependency on their husbands or because treatments were not covered by health insurance), pressure from relatives to remain married (or to keep problems to themselves), and threats from their partners. Some mothers mentioned that they had become accustomed to solving their own problems or that they were not yet ready for help (e.g., “As long as you are not ready to accept help, or you don’t find your own situation unacceptable, care providers can’t do anything”). In addition, mothers did not know anyone to whom they could turn or where they could go for help, and lacked information about their rights. This was especially true of immigrant mothers, although it was also the case for some of the native Dutch mothers, “I didn’t know where to go. I really didn’t know. Yes, now I know I can call a shelter . . . then I really had my hopes out for the people from the church.” (Dutch, 27, higher education)

Mothers were asked what could motivate other mothers in the same situation to seek help sooner. The mothers’ first advice was to talk about the violence sooner, even if it is difficult within their communities. They would also recommend becoming financially and socially independent—because this would help women to stand up to their violent partners or to terminate their relationships with them—and seeking formal or informal help that would be appropriate to their needs and situations. Mothers advised professionals to organize care in such a way that mothers would deal with only one professional (instead of several), who would help organize both practical help and parenting support. Furthermore, the mothers stressed that professionals should become better at detecting signals, and organize formal help in such a way that barriers to formal help are minimized, e.g., in cooperation with schools.

## Discussion

In this study we described the experiences of mothers of various ethnic backgrounds living in the Netherlands with parenting during and after IPV, their perceptions regarding the influence of IPV on their parenting, and their need for support in parenting and other areas. We also examined the ways in which parenting and need for support were related to ethnic background and the severity of IPV.

In line with findings from several previous studies (Hungerford et al. 2012; Levendosky et al. 2000), the majority of the mothers in this study reported negative experiences with parenting, both during and—though to a lesser extent—after the IPV period. At the time of the interview, more than a quarter of the mothers mentioned being unable to cope with parenting, either incidentally or continuously. In their narratives, there was a strong relationship between the IPV and parenting difficulties (in line with previous studies; Buehler and Gerard 2002; Krishnakumar and Buehler 2000; Letourneau et al. 2011). The severity of the IPV appeared to be related to more negative parenting experiences, and difficulty shielding children from the violence during the IPV period, as well as to parenting problems after the IPV period. In their narratives about how IPV influenced their parenting, mothers emphasized determinants similar to Belsky (1984): child and parent characteristics, sources of stress and support. They mentioned behavior problems of their children as a consequence of the IPV (remarkably mainly emphasized in the after IPV period), diminished personal wellbeing (e.g., mentally, physically, financially) and stress due to the IPV, as well as lack of support by the fathers (e.g., no involvement, undermining behavior, negative parenting practices, legal issues, problems with visiting arrangements), as well as by others in their informal network (especially during the IPV period).

Issues with fathers concerning co-parenting and custody have also been reported in other studies about parenting after divorce in IPV situations (Edleson and Williams 2007; Hardesty et al. 2008; Walker et al. 2004). Hardesty and Chung (2006) emphasize the impact of the dominant assumption (within both the legal system and public opinion) that maintaining a relationship with the father is in the best interest of the child. Because of this assumption, mothers’ attempts to protect themselves and their children are often overlooked and undermined, possibly increasing their fear of co-parenting. Our findings are comparable to these results. The mothers in our study reported feelings of being trapped between institutional demands to keep their children safe and the requirement to allow the children contact with their fathers. The limited previous studies that focus on fathering in IPV families confirm that father’s involvement and parenting skills in this context are reduced (Edleson and Williams 2007; Holden and Ritchie 1991).

The fact that mothers’ parenting problems decrease but do not disappear after IPV has ended might be explained by the prolonged impact of IPV on the wellbeing of both mothers and children. The finding that problems with children became more apparent after the IPV period might be related to the dominance of the violence during the IPV, which does not leave much room for sensitivity to the children’s problem signals.

In addition to negative effects of IPV, Levendosky and colleagues (2000) mention positive effects that IPV can have on the parenting of mothers. The mothers in the current study did not explicitly refer to any positive effects. They nevertheless mentioned that they had developed coping strategies for protecting their children from the IPV (or the negative consequences thereof). Strategies employed during the IPV period included avoiding quarrels, sending children away, paying them extra attention, and seeking external help. Strategies adopted after the IPV period included talking about the IPV, emphasizing that their children should not blame themselves, teaching their children that IPV is not normal, offering peace and structure, and stressing the importance of assertiveness in daughters and empathy and respect in sons. As the latter was not always modeled by the mothers and fathers, these goals will not necessarily be achieved (Parke 1996). Nevertheless, following Levendosky et al., such strategies can be classified as positive effects on parenting, in that mothers “mobilized their resources to respond to the violence on behalf of their children” (2000, p. 266).

We identified some differences in parenting practices (including coping strategies), specifically with regard to the period after the IPV. Turkish mothers were most likely to report negative parenting experiences. This finding might have been influenced by in-group collectivism and a lack of support. Within communities in which greater value is attached to family collectivism, there is a stronger tendency to endure violent relationships and to avoid airing one’s dirty laundry (see also Kasturirangan et al. 2004; Yerden 2008). This tendency was especially prevalent among the Turkish, Moroccan, and Hindustani-Surinamese mothers. This finding could be related to the stronger patriarchal culture and collectivism of these ethnic groups, as compared to the more individualistic or matriarchal nature of the Dutch, Afro-Surinamese, and Antillean groups (see also Merz et al. 2009). The Turkish community especially is known for its high level of collectivism and tight internal bonding (Gijsberts and Dagevos 2009). In addition, mothers’ history of immigration may affect the support they can invoke. A relatively high percentage of the Turkish mothers were new immigrants. Newly arrived mothers may have smaller networks and may be less informed about their rights as victims and available support services.

Other more general barriers to finding/seeking support that were mentioned by mothers included financial barriers, psychological barriers, and previous negative experiences. These findings are consistent with those reported by Rodriguez and colleagues (2009). Mothers eventually used multiple sources of informal and formal support. The majority of these mothers judged the support that they had received positively, but mixed or negative experiences were mentioned as well. Most of the mothers from all ethnic groups had experienced more positive support from their families after the IPV period than during this period. With regard to formal support, mothers were most negative about forced support, and they were most positive about easily accessible forms of parental support in places where they feel comfortable and know the people, e.g., support provided by schools and GPs.

More than half of the mothers were still in need of support at the time of the interview, especially with regard to practical help rebuilding their lives and support in raising their children (in that order). The order of these needs is consistent with Maslow’s (1954) hierarchy of needs. Instrumental help focused on basic needs (e.g., a place to live, income) is a necessary condition for enabling mothers to focus on parenting. This finding is also consistent with Letourneau and colleagues (2012), who emphasize the need for instrumental support in addition to other forms of support.

### Limitations

We would like to mention several limitations to this study. First, although we identified several tendencies, caution is required when interpreting and generalizing our findings due to some methodological limitations. The sample sizes of the ethnic groups were relatively small, so that data saturation could not be sufficiently established. In addition, it should be noted that the majority of the transcripts were not coded by multiple researchers; for this reason, inter-coder reliability could not be established for the majority of the transcripts. As described, at the start of the coding process, the coders discussed the codes and coding process extensively with the aim of reaching consensus.

Second, in this study we focused on the experiences of mothers, and we used their reports as sources of information about the children and their fathers (or stepfathers). Interviews with children and fathers might have revealed different perceptions (see Levendosky and Graham-Bermann 2001), as well as different or additional needs for support (see Hill and Jones 1997).

### Research and Practical Implications

Our study was based on a qualitative, cross-sectional design. Improving further understanding with regard to processes related to parenting and intergenerational violence will require longitudinal research. Longitudinal studies can provide additional insight into causal relationships, thus enhancing the available knowledge regarding the factors and mechanisms that deserve attention in the prevention and treatment of partner violence and its impact on children.

The results of our study do point to several opportunities for improving the situation and, more specifically, the parenting of families affected by IPV: (a) increasing public knowledge about IPV (thus also breaking the taboo and “culture of silence” regarding IPV), victims’ rights, and available assistance; (b) organizing easier access to informal and formal assistance; and (c) providing support at different levels, including customized parenting support.

The Dutch government has already started to increase knowledge about IPV, rights, and available assistance through national awareness campaigns (Ministerie van Justitie 2009). The scarce evidence shows that well-designed and tailored media campaigns might help to change attitudes within the general population, possibly having some effect on behavior (see Campbell and Manganello 2006). However, such campaigns might not reach all migrant families, because of such factors as their limited proficiency in Dutch and the predominant use of Dutch media (Van den Broek and Keuzenkamp 2008). In addition, national campaigns do not focus on the position of immigrant victims of IPV. We therefore recommend using additional media channels that are targeted toward immigrants or organizing community meetings for information and exchange (e.g., by immigrant self-help organizations).

Given that the mothers in this study often seek informal help first, increasing knowledge and facilitating communication within their families and communities might help to lift the taboo on family violence and also to enhance early detection and informal support. Community-based initiatives to support mothers (e.g., buddy projects, support groups, or internet platforms) should also be facilitated (see Sullivan et al. 2002). Self-help networks and organizations can be a particularly important safety net for women whose histories of violence have left them socially isolated, as well as for those who are unfamiliar with or distrust formal services (Lünnemann and Wijers 2010). These peer networks could both offer mutual aid and help bridge the gap with formal services. In addition, professional help should be based locally, at schools or other familiar and accessible locations. Furthermore, professional support should be tailored to the needs of mothers with a history of partner violence, as well as be culturally sensitive, taking into account the mothers’ own definition of problems and solutions. Support should be provided at different levels, although ideally the control should remain in the hands of one trusted professional. An important starting point is instrumental help that focuses on primary needs of mothers (e.g., housing, income), followed by help focused on their psychological wellbeing. Additionally, mothers should be supported with respect to their relationships with the fathers (including co-parenting arrangements), with a special focus on the safety of the children (possible forms include mediation, the Signs of Safety method developed by Turnell and Edwards (1999), and supervised visitation). Special attention should be paid to overcoming barriers, including the fear that the children will be removed from home or the contradictory demands placed on mothers to keep their children safe while allowing them to have contact with their fathers.

Internationally, systematic evidence-based interventions aimed at strengthening parenting in the context of family violence are scarce (Distelbrink et al. 2013). Based on this study, parenting support should focus on helping mothers to (re)create a healthy pedagogical family climate, to restore the appropriate roles in cases of role reversal, as well as to communicate with their children about their experiences. Communication about the violence (both during and after IPV) is important, as previous studies have shown that listening to children and involving them in finding solutions might help them to cope with their negative experiences (see Radford and Hester 2006), and might contribute to preventing aggressive behavior (Vetere and Cooper 2005). Finally, breaking the tendency toward subordination in daughters and toward machismo in sons can be an important aim of parenting support, which might help to prevent future violence.

## Footnotes

1 Although we acknowledge that fathers suffer as well and parenting support is at least as important for fathers who maintained contact with their children, this topic is beyond the scope of this article.

2 Interview questions are available from the corresponding author.

3 The classification could not be tested at the time of the study; the researchers are currently planning to do so.

4 More detailed information regarding the ways that mothers reported trying to help their children are available from the corresponding author.
